# Modulation Effects of Fe^3+^, Zn^2+^, and Cu^2+^ Ions on the Amyloid Fibrillation of α-Synuclein: Insights from a FTIR Investigation

**DOI:** 10.3390/molecules27238383

**Published:** 2022-12-01

**Authors:** Yan Li, Yang Yu, Gang Ma

**Affiliations:** Key Laboratory of Medicinal Chemistry and Molecular Diagnosis of Ministry of Education, Key Laboratory of Analytical Science and Technology of Hebei Province, College of Chemistry and Environmental Science, Hebei University, Baoding 071002, China

**Keywords:** amyloid, FTIR, α-synuclein, fibrillation, metal ion, fibril, secondary structure

## Abstract

Amyloid fibrillation of α-synuclein is implicated in the pathogenesis of Parkinson’s disease and heavy metal ions such as Fe^3+^, Zn^2+^, and Cu^2+^ are known to be involved in the process. In this work, we explored the use of FTIR spectroscopy to look into the modulation effects of Fe^3+^, Zn^2+^, and Cu^2+^ on the amyloid fibrillation of α-synuclein. We performed a curve-fitting analysis on the FTIR amide I bands of these α-synuclein fibril systems, namely, the pristine fibril and the fibrils prepared in the presence of Fe^3+^, Zn^2+^, and Cu^2+^. We found that the α-synuclein fibrils under the influences of metal ions all possessed a parallel β-sheet structure, turn structure, and disordered structure, similar to that of pristine α-synuclein fibril. We also observed metal-induced increases in the proportions of the β-sheet secondary structure within the α-synuclein fibrils, with Fe^3+^ being the most effective inducer. We performed second derivative analysis of the side chain carboxylic groups of α-synuclein fibrils and found that the side chain microenvironment of the α-synuclein fibrils was more influenced by Fe^3+^ than Zn^2+^, and Cu^2+^. In addition, our atomic force microscopic study revealed that the morphologies of α-synuclein fibrils under the influence of Fe^3+^ was quite different from that of the Zn^2+^ and Cu^2+^ systems. Our FTIR results suggested that the modulation effects of Fe^3+^, Zn^2+^, and Cu^2+^ on α-synuclein fibrillation occurred at both secondary and quaternary structural levels. At last, we proposed a mechanistic hypothesis to interpret how metal ions could affect the morphology of α-synuclein amyloid fibril based on the conformational plasticity properties of intrinsically disordered proteins.

## 1. Introduction

Parkinson’s disease is the second most common neurodegenerative disorder affecting aging populations worldwide [1]. Amyloid fibrillation of α-synuclein in brain tissues is the pathological hallmark of Parkinson’s disease [1,2]. Amyloid fibrillation is an interesting protein aggregation process in which proteins or peptides self-assemble into a fibrillar morphological structure. The length of the fibril can extend to several microns while the width of the fibril is usually in the nanoscale range [2]. At the molecular level, the very basic structure unit of an amyloid fibril is an extended β-sheet with its peptide strand running perpendicular to the fibril axis. This structure is called cross-β structure. Moreover, an amyloid fibril usually contains a paired β-sheet structure with the two β-sheets non-covalently bonded to each other through a steric-zipper configuration. Under X-ray diffraction, the amyloid structure will display a cross-β X-ray diffraction pattern which looks like two pairs of crescent moons oriented perpendicular to each other. The X-ray diffraction gives two characteristic reflections, ~4.8 Å and ~8–11 Å reflections. The former corresponds to the inter-strand distance between peptide strands and the latter corresponds to the inter-sheet distance between the paired β-sheets [3]. In vivo, amyloid fibrillation is devastating event and about 40 human diseases including Parkinson’s are closely linked to this phenomenon [2]. For Parkinson’s disease, amyloid fibril of α-synuclein or its related oligomeric precursors have been widely hypothesized to be the pathogenic agent [4].

Apparently, understanding the causes of amyloid fibrillation of α-synuclein and finding the modulation factors that can either accelerate or inhibit the aggregation process are very beneficial for the prevention and treatment of Parkinson’s disease. Many environment factors have been found to be able to affect the amyloid fibrillation of α-synuclein. Among them, heavy metal ions have been considered as important environmental factors to trigger the occurrence of amyloid fibrillation of α-synuclein. The reasons are as follows: first of all, as we know, α-synuclein is a soluble protein with 140 amino acid residues belonging to the intrinsically disordered protein family [5]. Its sequence can be divided into three parts with an amphipathic N-terminus (1–60), a hydrophobic central region known as NAC (61–95), and an acidic C-terminal region (96–140) [6]. The carboxylic functional groups of aspartic acid and glutamic acid residues of α-synuclein are highly prone to be bound by heavy metal ions and other residues such as methionine, asparagine, and histidine can also be involved in the binding process [7]. The binding events could affect the amyloid fibrillation of α-synuclein at both the secondary and quaternary structural levels. Secondly, epidemiological evidence indicated the occupational exposure to heavy metals might increase the risk of Parkinson’s disease [8]. There is also evidence to show that altered metal homeostasis might be involved in the progression of neurodegenerative diseases. Indeed, in the brains of Parkinson’s disease patients, higher concentrations of heavy metal ions such as copper, iron, zinc, and aluminum have been detected in the cerebrospinal fluid or the substantia nigra [9].

The possible role of heavy metal ions in the etiology of Parkinson’s disease has sparked great interest among scientists to investigate the modulation effects of metal ions on the amyloid fibrillation of α-synuclein. For example, Uversky and coworkers performed the pioneering work about the effects of 15 metal ions on the amyloid fibrillation of α-synuclein. Their work focused on the metal ion effects on the aggregation kinetics and pathway of α-synuclein. These metal ions include mono-, di-, and trivalent metal ions such as Li^+^, K^+^, Na^+^, Cs^+^, Ca^2+^, Co^2+^, Cd^2+^, Cu^2+^, Fe^2+^, Mg^2+^, Mn^2+^, Zn^2+^, Co^3+^, and Al^3+^. Their results demonstrated that all of these metal ions can accelerate the amyloid fibrillation of α-synuclein. They further showed that on the aggregation pathway, the natively unfolded α-synuclein adopted a partially folded conformation under the influence of various metal cations [9]. Rao investigated the different effects of Fe^3+^ and Cu^2+^ on the morphological changes of the amyloid fibrils of α-synuclein as well as its three mutant variants (A30P, A53T, and E46K) with transmission electron microscopy (TEM) [10]. They illustrated the metal-specific fibril morphology phenomenon. They found that Cu^2+^ could lead to thin and long fibrils while the fibril under the presence of Fe^3+^ displayed short and thick features. Kim and coworkers chose Ca^2+^ as a representative hard divalent metal ion and investigated its effect on the amyloid fibrillation of α-synuclein using a variety of biophysical tools such as ion mobility-mass spectrometry (IM-MS), synchrotron small-angle X-ray scattering (SAXS), TEM [11]. They found that the binding of Ca^2+^ onto α-synuclein monomers induced the formation of an extended protein conformation which promoted the rapid fibrillation. They further showed that Ca^2+^ induced the formation of interfibrillar aggregates due to the enhanced electrostatic and hydrophobic interactions. Lorentzon et al. also investigated the differential effects of Cu^2+^ and Fe^3+^ ions on in vitro amyloid formation of α-synuclein. They found mutation as well as acetylation could weaken the acceleration effects of metal ions on the amyloid formation of α-synuclein [12]. Bai et al. found the trivalent metal ions (lanthanides) could accelerate the amyloid fibrillation of α-synuclein faster than divalent cations and they also used NMR to determine the binding sites of La^3+^ ions on α-synuclein [13]. Lingor et al. proposed that using a chelator could be an effective way to contain the deleterious effects of elevated metal levels in the brain by inhibiting the interaction between α-synuclein and metal interaction [14].

Despite this progress, how the metal ions affect the structures of α-synuclein amyloid fibrils at both the secondary and quaternary structural levels are largely lacking. In this regard, we explored the use of Fourier-transform infrared (FTIR) spectroscopy and atomic force microscopy (AFM) to investigate the modulation effects of Fe^3+^, Zn^2+^, and Cu^2+^ on the amyloid fibrillations of α-synuclein. FTIR spectroscopy is an ideal tool to look into the molecular level structure of amyloid fibril [15,16,17,18,19,20,21,22]. With FTIR spectroscopy, we could extract the secondary structure information of an amyloid fibril by analyzing the protein amide I FTIR band (1700–1600 cm^−1^). Moreover, by analyzing the feature bands of amino acid side chains, e.g., the carboxylic group, we were able to look into the quaternary structural information of an amyloid fibril. AFM investigation helps us to look into the morphological changes of α-synuclein fibrils induced by metal ions. We also proposed a hypothesis to address how metal ions could affect the morphology of α-synuclein amyloid fibril based on the conformational plasticity property of intrinsically disordered protein. To the best of our knowledge, a thorough FTIR investigation on the modulation effects of Fe^3+^, Zn^2+^, and Cu^2+^ on the amyloid fibrillations of α-synuclein has not been reported. We hope our work can provide some new insights into the effects of metal ions on α-synuclein amyloid fibrillation.

## 2. Results and Discussion

We first used the FTIR curve-fitting method in the amide I region to address the effects of the three metal ions on the amyloid fibril of α-synuclein at the secondary structural level. It is well known that curving-fitting on the amide I region of a protein FTIR spectrum can resolve different subpeaks with each subpeak corresponding to each type of secondary structure. According to the summary made in the seminal review by Barth et al., each type of secondary structure such as β-sheet structure, turn structure, and disordered structure displays its characteristic frequency in the amide I region in the protein FTIR spectrum [23]. Curve-fitting can give us not only the actual frequency of each subpeak, but also give us the proportion of the relative area of each subpeak [24,25,26,27,28,29,30,31]. 

**Figure 1:**
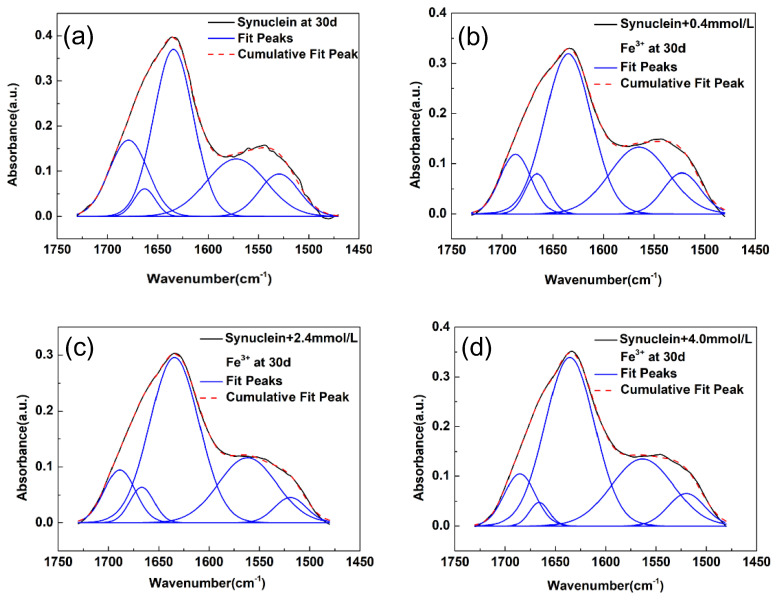
FTIR spectra of α-synuclein amyloid fibril under the influences of different concentrations of Fe^3+^ and curve-fitting results in the amide I region. (**a**) control; (**b**) 0.4 mM; (**c**) 2.4 mM; (**d**) 4.0 mM; solid black line: measured spectrum; dash red line: overall fit; solid blue line: fitted subpeak. a.u.: arbitrary unit.

In Figure 1a, the FTIR spectrum of the control system, i.e., the pristine α-synuclein amyloid fibril, was first analyzed with the curve-fitting method. The curve-fitting was performed in the spectral region of 1725–1475 cm^−1^ which covers both the amide I region (1700–1600 cm^−1^) and the amide II region (1600–1500 cm^−1^). As we can see, in the amide I region, three subpeaks were clearly resolved. This suggests that α-synuclein fibril possesses three major secondary structures. The three subpeaks are located at 1634 cm^−1^, 1662 cm^−1^, and 1678 cm^−1^, respectively. They corresponded to three secondary structures, namely, parallel β-sheet, disordered structure, and turn structure [18,26]. There are also two additional resolved peaks in the curve-fitting in the amide II region. The two subpeaks in the amide II region were due to the complicated absorptions of amino acid side chains as well as the absorption of amide II vibrations in this spectral region [26]. The detailed curve-fitting results including the fitted frequencies and relative areas of the three subpeaks were listed in Table 1. 

The secondary structural feature revealed by curve-fitting results is consistent with the previous structural model of α-synuclein amyloid fibril by cryo-EM. In such cryo-EM structural model, the amyloid fibril of α-synuclein consists of pairs of protofibrils or filaments. The protofibril is the basic structural unit of mature α-synuclein fibril. The protofibril is like a column of piled α-synuclein monomers with each monomer being in a “Greek key-like” conformation [32,33]. These monomers were bound together through the typical parallel β-sheet configuration. In the “Greek key-like” conformation, each α-synuclein monomer has three different types of secondary structures, parallel β-sheet, disordered structure, and turn structure. This structural feature of α-synuclein amyloid fibril is exactly what had been revealed by the curve-fitting shown in Figure 1a.

We now look into the curve-fitting results of α-synuclein amyloid fibril formed under the influence of metal ion of Fe^3+^. As shown in Figure 1b–d, the FTIR spectra of α-synuclein amyloid fibrils in the spectral region of 1725–1475 cm^−1^ displayed some spectral changes under the influences of three different concentrations of Fe^3+^. To look into the spectral variations in a quantitative way, we also performed curve-fitting analysis on these FTIR spectra. During curve-fitting, the initial estimates for the numbers and frequencies of subpeaks were obtained through second derivative technique and the corresponding second derivative spectra were shown in Figures S6–S9. The curve-fitting results were listed in Table 1. As can see from the curve-fitting results, all of the three synuclein-Fe^3+^ systems displayed the presence of three secondary structures, similar to that in the pristine α-synuclein amyloid fibril system. The three subpeaks are located at ~1634 cm^−1^, ~1665 cm^−1^, and ~1686 cm^−1^, respectively. They were assigned to parallel β-sheet, disordered structure, and turn structure, respectively. Yet, if we look into the curve-fitting data in details, one obvious change is that the proportion of the β-sheet structure in the α-synuclein amyloid fibril under the influence of Fe^3+^ increased with the increase in Fe^3+^ concentration, and a 24% increase (refer to Table S1) was observed in the presence of 4.0 mM Fe^3+^. In summary, the curve-fitting results in Figure 1 revealed a modulation effect of Fe^3+^ on the amyloid fibril of α-synuclein at the secondary structural level. 

**Figure 2:**
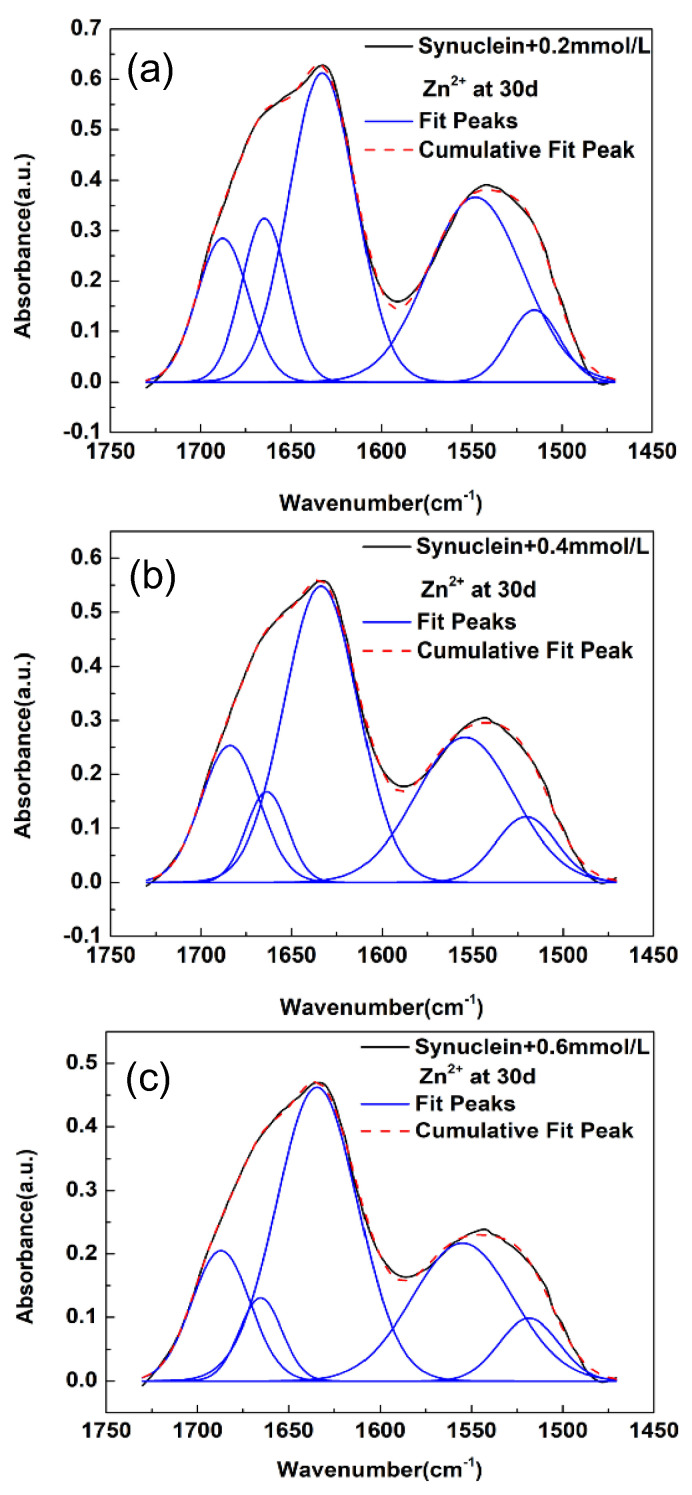
FTIR spectra of α-synuclein amyloid fibril under the influences of different concentrations of Zn^2+^ and curve-fitting results in the amide I region. (**a**) 0.2 mM; (**b**) 0.4 mM; (**c**) 0.6 mM; solid black line: measured spectrum; dash red line: overall fit; solid blue line: fitted subpeak. a.u.: arbitrary unit.

We now look into the curve-fitting results of α-synuclein amyloid fibril formed under the influence of metal ion of Zn^2+^. As shown in Figure 2a–c, the FTIR spectra of α-synuclein amyloid fibrils in the spectral region of 1725–1475 cm^−1^ displayed some spectral changes under the influences of three different concentrations of Zn^2+^. We also performed curve-fitting analysis on these FTIR spectra. During curve-fitting, the initial estimates for the numbers and frequencies of subpeaks were obtained through second derivative analysis on these FTIR spectra which were shown in Figures S10–S12. As shown in Figure 2 and Table 2, all of the three synuclein-Zn^2+^ systems displayed the presence of three secondary structures (i.e., parallel β-sheet, disordered structure, turn structure), similar to that in the pristine α-synuclein system and the synuclein-Fe^3+^ system. The three subpeaks are located at ~1634 cm^−1^, ~1665 cm^−1^, and ~1686 cm^−1^, respectively. The detailed information about the frequencies and relative areas corresponding to the three secondary structures were also listed in Table 2. As we can see, the proportions of the relative areas of the β-sheet structure increased to some extent with the increase in Zn^2+^ concentration. Yet, the increase in the proportions of the relative areas of the β-sheet structure in the synuclein-Zn^2+^ system is less significant than in the synuclein-Fe^3+^ system (also refer to Tables S1 and S2). Overall, the curve-fitting results in Figure 2 suggest that the modulation effects of Zn^2+^ on the amyloid fibril of α-synuclein at the secondary structural level were moderate, not as significant as that of Fe^3+^.

**Figure 3:**
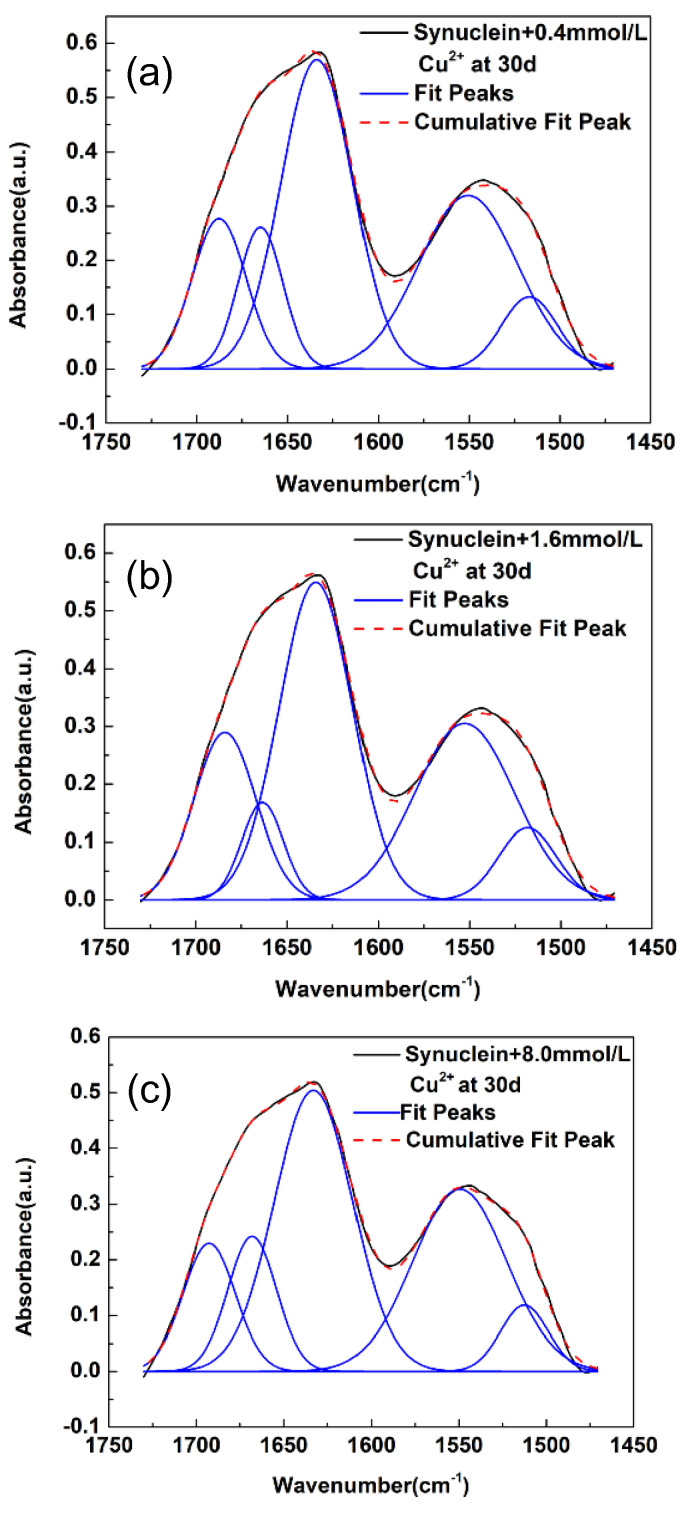
FTIR spectra of α-synuclein amyloid fibril under the influences of different concentrations of Cu^2+^ and curve-fitting results in the amide I region. (**a**) 0.4 mM; (**b**) 1.6 mM; (**c**) 8.0 mM; solid black line: measured spectrum; dash red line: overall fit; solid blue line: fitted subpeak. a.u.: arbitrary unit.

Next, we look into the curve-fitting results of synuclein amyloid fibril formed under the influence of metal ion of Cu^2+^. As shown in Figure 3a–c, the FTIR spectra of α-synuclein amyloid fibrils in the spectral region of 1725–1475 cm^−1^ displayed some spectral changes under the influences of three different concentrations of Cu^2+^. We also performed curve-fitting analysis on these FTIR spectra. During curve-fitting, the initial estimates for the numbers and frequencies of subpeaks were obtained through second derivative analysis on these FTIR spectra which were shown in Figures S13–S15. As shown in Figure 3 and Table 3, all of the three synuclein-Cu^2+^ systems displayed the presence of three secondary structures (i.e., parallel β-sheet, disordered structure, turn structure), similar to that in the pristine α-synuclein system, the synuclein-Fe^3+^ system, and the synuclein-Zn^2+^ system. The three subpeaks are located at ~1634 cm^−1^, ~1665 cm^−1^, and ~1686 cm^−1^, respectively. The detailed frequencies and relative areas corresponding to the three secondary structures were listed in Table 3. As we can see, the proportions of the relative areas of the β-sheet structure increased to some extent with the increase in Cu^2+^ concentration. Yet, the increase in the proportions of the relative areas of the β-sheet structure in the synuclein-Cu^2+^ system is less significant than in the synuclein-Fe^3+^ system (also refer to Tables S1 and S3). Overall, the curve-fitting results in Figure 3 suggest that the modulation effects of Cu^2+^ on the amyloid fibril of α-synuclein at the secondary structural level were moderate, not as significant as that of Fe^3+^ and similar to that of Zn^2+^.

By comparing the three α-synuclein systems under the influences of the three metal ions, we can conclude that all of the three metal ions displayed the modulation effects on the secondary structures of α-synuclein amyloid fibrils were. All of the three amyloid fibril systems showed increased β-sheet secondary structures due to the modulation effects of metal ions and Fe^3+^ appeared to be the most effective secondary structural modulator. In addition, we do observe that the FTIR frequencies of the turn structures in the α-synuclein amyloid fibrils under the influence of metal ions were higher than that of pristine α-synuclein amyloid fibrils.

**Figure 4:**
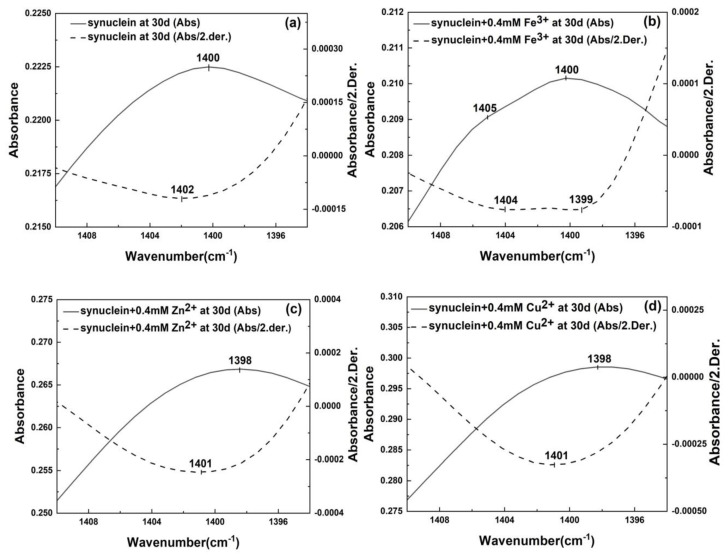
FTIR spectra of α-synuclein amyloid fibril under the influences of different metal ions and their corresponding second derivative spectra in the COO^−^ symmetric stretching region. (**a**) control; (**b**) Fe^3+^; (**c**) Zn^2+^; (**d**) Cu^2+^; solid line: measured spectrum; dash line: second derivative.

We now look into the modulation effects of metal ions on the α-synuclein amyloid fibril at the quaternary structure level. The quaternary structure of amyloid fibril in this work refers to the structural details of the amino acid side chains of α-synuclein within an amyloid fibril. As we mentioned before, mature α-synuclein amyloid fibril consists of associated protofibril units. During amyloid fibrillation, the amino acid side chains played important roles in driving the association of protofibrils to form mature amyloid fibril. α-Synuclein has a large number of carboxylic groups in its amino acid side chains. These carboxylic groups could be the binding sites for metal ions. In the following section, we will use FTIR to see whether we can obtain spectral evidences to support the presence of the interaction between metal ion and the carboxylic side chain group within the amyloid fibril of α-synuclein. The carboxylic side chain group has two IR absorptions in the IR fingerprint region. One is located at around 1570–1556 cm^−1^, corresponding to the asymmetric stretch of COO^−^; the other is located at around 1400 cm^−1^, corresponding to the symmetric stretch of COO^−^ [23]. The absorption of the asymmetric stretch of COO^−^ overlaps with the amide II absorption of the protein backbone and it is thus difficult to be analyzed [23]. Fortunately, the symmetric stretch of COO^−^ is located in a relatively clean region around 1400 cm^−1^ and we will focus on its spectral variations under the influences of metal ions. Figure 4 showed the FTIR spectra of α-synuclein amyloid fibrils under the influences of different metal ions in the COO^−^ symmetric stretching region of 1410–1390 cm^−1^. The dashed lines were the corresponding second derivative spectra. Second derivative technique is a resolution enhancement technique which could help us resolve the hidden spectral details under a seemingly featureless spectral envelop of a FTIR absorption spectrum. As we can see in Figure 4a, the pristine α-synuclein amyloid fibril displayed a single resolved peak at 1402 cm^−1^ in its second derivative spectrum; the α-synuclein amyloid fibril under the influence of Fe^3+^ displayed two resolved peaks at 1404 cm^−1^ and 1399 cm^−1^ in its second derivative spectrum; the α-synuclein amyloid fibril under the influence of Zn^2+^ displayed a single resolved peak at 1401 cm^−1^ in its second derivative spectrum; the α-synuclein amyloid fibril under the influence of Cu^2+^ also displayed a single resolved peak at 1401 cm^−1^ in its second derivative spectrum. The results in Figure 4 indicated that (1) Fe^3+^ has the most significant effect on the symmetric stretching band of COO^−^ as more subpeaks were resolved by second derivative treatment as compared with the control as well as synuclein-Zn^2+^ and synuclein-Cu^2+^ systems; (2) both Zn^2+^ and Cu^2+^ caused a slight red-shift of COO^−^ symmetric stretching band as compared with the COO^−^ symmetric stretching band of the pristine α-synuclein amyloid fibril. These observations provided the evidence for the presence of the interaction between metal ions and the COO^−^ side chain groups of α-synuclein. It further supports the argument that the metal ions of Fe^3+^, Zn^2+^, and Cu^2+^ had their modulation effects on α-synuclein fibrillation at the quaternary structural level.

**Figure 5:**
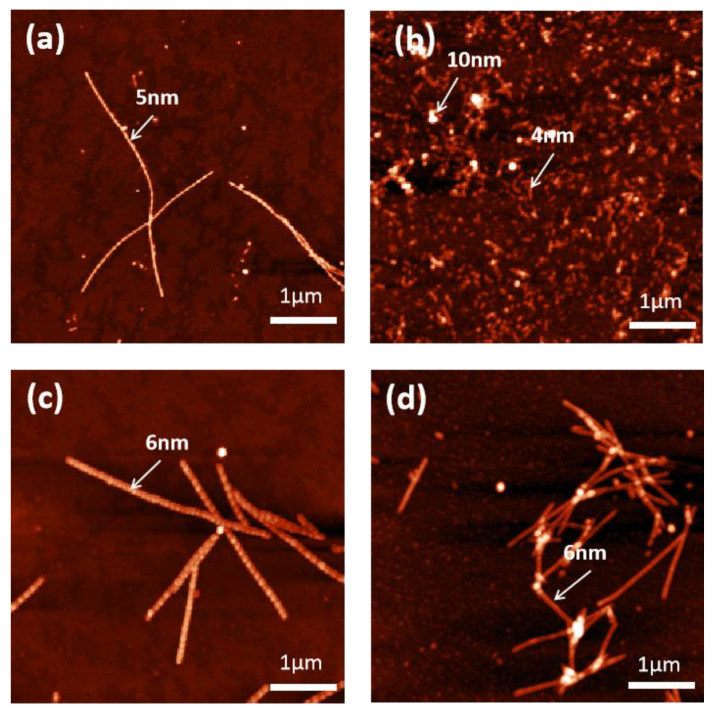
AFM results of the effects of Fe^3+^, Zn^2+^, and Cu^2+^ on the morphology of α-synuclein amyloid fibril. (**a**) control; (**b**) 0.4 mM Fe^3+^; (**c**) 0.4 mM Cu^2+^; (**d**) 0.4 mM Zn^2+^.

We now look into the effect of metal ions on the morphology of α-synuclein amyloid fibril using AFM. As we can see in Figure 5 as well as Figure S16, compared with the pristine amyloid fibril of α-synuclein (i.e., the control), the Fe^3+^ system displayed the most significant morphological changes. Fe^3+^ changed the fibril from a relatively rigid morphology to a “worm-like” morphology. The height of the control shown in Figure 5a was about 5 nm for the control. Yet, the height of the amyloid fibril in the Fe^3+^ system shown in Figure 5b was ~4–10 nm. In contrast, Zn^2+^ and Cu^2+^ did not change the morphology of α-synuclein amyloid fibril significantly. It seemed that the fibril heights in Zn^2+^ and Cu^2+^ increased to some extent and the fibrils appeared more rigid compared with the control. The observed different effects between Fe^3+^ and Zn^2+^/Cu^2+^ on the morphology of α-synuclein amyloid fibril were consistent with the FTIR results revealed in Figure 1, Figure 2, Figure 3 and Figure 4. In the following, we proposed a hypothesis to interpret the morphological effect of metal ions based on the conformational plasticity property of intrinsically disordered protein.

**Figure 6:**
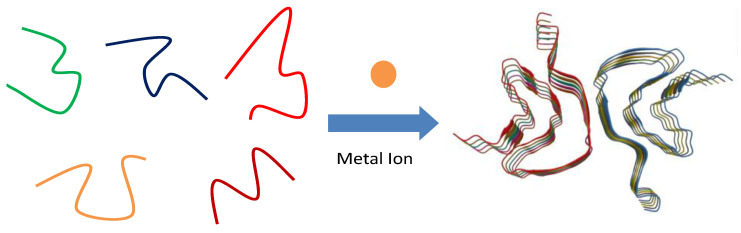
Modulation effect of metal ion on the morphology of α-synuclein through conformational plasticity mechanism.

Figure 6 is an illustration of a proposed hypothesis suggesting how metal ions could affect the morphology of α-synuclein. On the right part of Figure 6, we can see the supramolecular structure of α-synuclein amyloid fibril. This model is based on previously published cryo-EM results (PDB ID: 6CU7) and it is only for illustrative purpose in this work [33]. In this model structure, α-synuclein amyloid fibril consists of two protofibril units and each unit consists of five α-synuclein monomers in “Greek key-like” conformation. The “Greek key-like” conformation (i.e., the secondary structure) of the α-synuclein monomer not only could affect the binding configuration between monomers along the fibril axis, but also could influence the contour shape of the binding surface between the two protofibrils. This will eventually have a significant impact on the final morphology of α-synuclein amyloid fibril. As we mentioned above, α-synuclein is an intrinsically disordered protein. An important property of intrinsically disordered protein is that the protein displays conformational plasticity [34]. In other words, an intrinsically disordered protein has no fixed conformation. Rather, it changes its conformation from time to time. Therefore, an intrinsically disordered protein always samples a complex mixture of conformations as shown in the left part of Figure 6, including partially foldable, potentially foldable, differently foldable, or completely unfoldable peptide backbone conformations. 

The free energy landscape of an intrinsically disordered protein is thus relatively flat and simple, lacking a deep energy minimum that is always found in ordered proteins. Such a simplified and flattened energy landscape makes the conformation of intrinsically disordered protein very sensitive to environmental changes (e.g., metal ion presence). This means that when metal ion is present in the solution, specific conformations of α-synuclein monomers could be induced due to the interaction between metal ion and α-synuclein (either through electrostatic interaction or through specific binding) [35]. The metal ion induced α-synuclein conformations will be passed down to the following-up amyloid fibrillation processes such as fibril nucleation and fibril growth and eventually affect the actual shape of the “Greek key-like” monomer and then the morphology of α-synuclein amyloid fibril. As for the observed morphological differences between synuclein-Fe^3+^ system and synuclein-Zn^2+^/synuclein-Cu^2+^ systems, it is likely due to the fact that the Fe^3+^ ion carrying three charges can lead to a stronger electrostatic interaction between α-synuclein and metal ion than the Zn^2+^/Cu^2+^ ion carrying two charges. Such an increased electrostatic interaction between α-synuclein and Fe^3+^ ion might further boost the packing of the conformation of α-synuclein, making Fe^3+^ ion display a strong modulation effect on the conformation plasticity of α-synuclein monomer.

## 3. Materials and Methods

### 3.1. Materials

α-Synuclein was expressed with *E. coli* system and the procedure was described in the following section. Sodium chloride (NaCl) with >99% purity was purchased from Merck (Saint Louis, MO, USA). Ferric chloride (FeCl_3_), cupric chloride (CuCl_2_), and zinc chloride (ZnCl_2_) in analytical grade were all obtained from Tianjin Guangfu (Tianjin, China). Sodium azide (NaN_3_) with a purity of 99% was purchased from Tianjin Chemical Plant (Tianjin, China). Sodium phosphate dibasic dehydrate (Na_2_HPO_4_·2H_2_O) with >99% purity and sodium dihydrogen phosphate dehydrate (NaH_2_PO_4_·2H_2_O) with >99% purity was purchased from Merck (Saint Louis, MO, USA). Potassium phosphate monobasic (KH_2_PO_4_) (99%) was purchased from Aladdin (Shanghai, China). Potassium chloride (KCl) with >99% purity was purchased from Tianjin Kemiou Chemical Reagent Co., Ltd. (Tianjin, China). Ammonium sulfate ((NH_4_)_2_SO_4_) with >99% purity was purchased from Tianjin Guangfu (Tianjin, China). Glycerol (AR grade) was purchased from Tianjin Damao Chemical Reagent Factory. (Tianjin, China). Coomassie brilliant blue R-250 was purchased from Tianjin Guangfu (Tianjin, China). Ampicillin Na (AMP), isopropyl β-D-1-thiogalactopyranoside (IPTG) and phenylmethanesulfonyl fluoride (PMSF) with >99% purity was purchased from Biotopped Life Sciences through a local vendor (Baoding, China). Trizma base with >99% was purchased from Merck (Saint Louis, MO, USA). No further purification was performed on all the chemicals.

### 3.2. Cloning, Expression, and Purification of α-Synuclein

α-Synuclein used in this work was expressed in our own lab. By referring to previous works [22,36,37,38], we had developed the following protocol to express α-synuclein. For bacterial protein expression, the construct was transformed into *E. coli* BL21 (DE3) (TransGen Biotech, Beijing, China) and grown on an AMP plate. A single colony was inoculated, amplified, and grown to 4 × 250 mL LB culture containing 100 μg/mL AMP under shaking at 220 rpm at 37 °C. The protein expression was induced by 120 μg/mL IPTG. The cells were harvested 5 h after induction when OD600 reached 0.6. The solution was then centrifuged at 11,000 rpm (8400 rcf) for 5 min at 4 °C. The cell pellet was re-suspended in 100 mL sterile PBS buffer at pH 7.4 followed by adding 175 μg/mL PMSF. The cell pellet was kept at −80 °C for 1 h. The solution was thawed in water bath at 37 °C. The solution was incubated on ice for 30 min. Cells were lysed through sonication for 5 min followed by 10 min centrifugation at 11,000 rpm (8400 rcf). The sonication was performed on ice using a probe sonicator (Model: JY92-IIDN) from SCIENTZ (Ningbo, China) with a working frequency of 25 kHz and a working power of 40 W. The sonication cycle is 10 s operation time with 5 s waiting time. The resultant suspension became more transparent and less viscous as cells were lysed. Then, 40% (NH4)_2_SO_4_ was added into this suspension under stirring on ice bath for 10 min followed by 30 min centrifugation at 11,000 rpm (8400 rcf). The resultant pellet was re-suspended in 10 mL PBS at pH 7.4. Next, the re-suspended pellet was placed in a dialysis tubing with MWCO of 14,000 and was extensively dialyzed against an equilibrating buffer consisting of 20 mM NaCl, 20 mM Tris-HCl at pH 8.0, 5% glycerol, at 4 °C for 24 h. The dialyzed α-synuclein was removed from the dialysis tubing and centrifuged for 5 min at 12,000 rpm (10,000 rcf) to precipitate denatured contaminants. The supernatant was filtered through 0.22 μm filter membrane before loading onto a DEAE-Sepharose column. To purify the protein, 20 mM Tris-HCl buffer at pH 8 with a salt gradient from 100 to 600 mM NaCl was used on the dialyzed α-synuclein. A-synuclein was eluted at approximately 300 mM NaCl. Fractions containing α-synuclein were pooled and dialyzed extensively at 4 °C against deionized water. The elution fractions containing α-synuclein were confirmed by 15% SDS-PAGE and pooled together. A >95% purity was estimated through SDS-PAGE (Figure S1 in the Supplementary Materials) and the correct molecular weight (MW: 14460) of α-synuclein was confirmed by mass spectrometry (Figure S2). The obtained α-synuclein were lyophilized and stored at −80 °C until further use.

### 3.3. Amyloid Fibrillation Conditions

A 4 mg/mL α-synuclein incubation solution was prepared in a 10 mM Tris-HCl buffer at pH 7.4 and the incubation solution also contained 140.0 mM NaCl and 200 ppm NaN_3_. The solution was incubated at 37 °C at 1000 rpm. When investigating the effects of metal ions, the incubation solutions contained three different concentrations of FeCl_3_ or CuCl_2_ or ZnCl_2_. We used 2 mL HPLC sampling vial (HM-0712) which has a cap with inside Teflon-lining from HAMAG (Ningbo, China) to hold the incubation solution. This type of vial has a good sealing to prevent evaporation. A thermal shaker (MS-100) from Allsheng (Hangzhou, China) was used as incubator. The incubation last for 30 days to ensure the amyloid fibrillation of α-synuclein to reach its equilibrium phase. The incubation was monitored using thioflavin T (ThT) fluorescence assay and the obtained fibrillation kinetic results were shown in Figures S3–S5. At the end of incubation, a small aliquot of the incubation solution was sampled for atomic force microscopy (AFM) measurement. The rest of the incubation solution was collected. The amyloid fibrils inside the incubation solution were harvested through centrifugation and then lyophilized for further FTIR measurement.

### 3.4. FTIR Spectroscopy 

The FTIR spectra were performed with a Vertex 70 FTIR spectrometer (Bruker, Ettlingen, Germany). The attenuated total reflection (ATR) mode was used to measure the FTIR spectrum of the sample. A Pike Technologies MIRacle single-reflection ATR accessory (Madison, WI, USA) with a diamond element was used. Spectra were collected with 4 cm^−1^ resolution and 128 scans. A factor of 4 was used for zero filling. FTIR data processing was performed with Bruker OPUS (8.7) software. Curve-fitting of FTIR spectra was performed with Origin (8.1) software. The raw FTIR spectrum was first processed through second derivative treatment to determine the initial estimates for subpeak positions and subpeak number within the spectral region of 1725–1475 cm^−1^; then the spectrum was curve-fitted to resolve the subpeaks under the spectral envelop using the build-in function of Origin.

### 3.5. AFM Characterization

The morphology of amyloid fibril of α-synuclein was studied by AFM (NT-MDT, Zelenograd, Russia). The silicon cantilevers were also purchased from NT-MDT with a resonance frequency of 100 kHz and a nominal force constant of 3 N/m. The AFM sample was prepared on freshly cleaved mica. A 2.5 μL aliquot of the sample was diluted first with 500 μL water from a Millipore system (Billerica, MA, USA). Then, 10 μL of the diluted sample was dropped onto the mica surface. The mica surface was further dried with a nitrogen flow.

## 4. Conclusions

In summary, we performed a thorough FTIR investigation on the modulation effects of Fe^3+^, Zn^2+^, and Cu^2+^ on the amyloid fibrillation of α-synuclein. Our FTIR spectral analysis indicated that α-synuclein amyloid fibril possessed a parallel β-sheet structure, turn structure, and disordered structure. Our FTIR spectral analysis further suggested that the modulation effects of Fe^3+^, Zn^2+^, and Cu^2+^ on α-synuclein fibrillation occurred at both secondary and quaternary structural levels, with Fe^3+^ being the most effective modulator. Furthermore, we proposed a mechanistic hypothesis to interpret how metal ions could affect the morphology of α-synuclein amyloid fibril based on the conformational plasticity property of intrinsically disordered protein. We hope our work could shed some new light on the complex effects of heavy metal ions on the amyloid fibrillation of α-synuclein.

## Figures and Tables

**Table 1:** Curve-fitting results of the FTIR spectra of α-synuclein amyloid fibril under the influence of different concentrations of Fe^3+^.

Fe^3+^ (mM)	Fitted Frequency (cm^−1^)	Subpeak Assignment	Relative Area (%)
0	1634	β-sheet	39.5
	1662	disordered	21.7
	1678	turn	38.8
0.4	1634	β-sheet	46.1
	1665	disordered	23.2
	1686	turn	30.7
2.4	1634	β-sheet	47.0
	1666	disordered	23.0
	1688	turn	30.0
4.0	1635	β-sheet	49.0
	1666	disordered	20.2
	1685	turn	30.8

**Table 2:** Curve-fitting results of the FTIR spectra of α-synuclein amyloid fibril under the influence of different concentrations of Zn^2+^.

Zn^2+^ (mM)	Fitted Frequency (cm^−1^)	Subpeak Assignment	Relative Area (%)
0.2	1632	β-sheet	40.8
	1664	disordered	27.7
	1687	turn	31.5
0.4	1633	β-sheet	42.6
	1663	disordered	27.9
	1683	turn	33.5
0.6	1634	β-sheet	44.5
	1665	disordered	23.5
	1687	turn	32.0

**Table 3:** Curve-fitting results of the FTIR spectra of α-synuclein amyloid fibril under the influence of different concentrations of Cu^2+^.

Cu^2+^ (mM)	Fitted Frequency (cm^−1^)	Subpeak Assignment	Relative Area (%)
0.4	1633	β-sheet	42.0
	1664	disordered	26.3
	1687	turn	31.7
1.6	1634	β-sheet	42.5
	1663	disordered	23.6
	1684	turn	33.9
8.0	1632	β-sheet	43.6
	1668	disordered	27.2
	1692	turn	29.2

## Data Availability

The data presented in this study are available on request from the corresponding author.

## Supplementary Materials

The following supporting information can be downloaded at: https://www.mdpi.com/article/10.3390/molecules27238383/s1, Figure S1: SDS-PAGE result of α-synuclein; Figure S2: Mass spectrometry (MS) result of α-synuclein; Figure S3: Fibrillation kinetics of α-synuclein under different concentrations of Fe^3+^ by ThT fluorescence assay; Figure S4: Fibrillation kinetics of α-synuclein under different concentrations of Zn^2+^ by ThT fluorescence assay; Figure S5: Fibrillation kinetics of α-synuclein under different concentrations of Cu^2+^ by ThT fluorescence assay; Figure S6: Second derivative spectrum of α-synuclein amyloid fibril in the amide I region; Figure S7: Second derivative spectrum of α-synuclein amyloid fibril under the influence of 0.4 mM Fe^3+^ in the amide I region; Figure S8: Second derivative spectrum of α-synuclein amyloid fibril under the influence of 2.4 mM Fe^3+^ in the amide I region; Figure S9: Second derivative spectrum of α-synuclein amyloid fibril under the influence of 4.0 mM Fe^3+^ in the amide I region; Figure S10: Second derivative spectrum of α-synuclein amyloid fibril under the influence of 0.2 mM Zn^2+^ in the amide I region; Figure S11: Second derivative spectrum of α-synuclein amyloid fibril under the influence of 0.4 mM Zn^2+^ in the amide I region; Figure S12: Second derivative spectrum of α-synuclein amyloid fibril under the influence of 0.6 mM Zn^2+^ in the amide I region; Figure S13: Second derivative spectrum of α-synuclein amyloid fibril under the influence of 0.4 mM Cu^2+^ in the amide I region; Figure S14: Second derivative spectrum of α-synuclein amyloid fibril under the influence of 1.6 mM Cu^2+^ in the amide I region; Figure S15: Second derivative spectrum of α-synuclein amyloid fibril under the influence of 8.0 mM Cu^2+^ in the amide I region; Figure S16: The height analysis of amyloid fibrils of the pristine α-synuclein system, α-synuclein+Fe^3+^ system, α-synuclein+Zn^2+^ system, and α-synuclein+Cu^2+^ system; Table S1: The proportion increase of β-sheet secondary structure in α-synuclein amyloid fibril due to the influences of different concentrations of Fe^3+^; Table S2: The proportion increase of β-sheet secondary structure in α-synuclein amyloid fibril due to the influences of different concentrations of Zn^2+^; Table S3: The proportion increase of β-sheet secondary structure in α-synuclein amyloid fibril due to the influences of different concentrations of Cu^2+^.
