# Supplementary material for: Modulation Effects of Fe3+, Zn2+, and Cu2+ Ions on the Amyloid Fibrillation of α-Synuclein: Insights from a FTIR Investigation
Source: Molecules. 2022 Dec 1;27(23):8383. doi: 10.3390/molecules27238383 (PMC9740228; doi:10.3390/molecules27238383)
Supplement: Supplementary file 1 [file molecules-27-08383-s001.zip › molecules-1998023-supplementary.pdf]

Supplementary Materials

Modulation Effects of Fe^{3+}, Zn^{2+}, and Cu^{2+} Ions on the Amyloid Fibrillation of α-Synuclein: Insights from a FTIR Investigation

Yan Li [†], Yang Yu [†], and Gang Ma *

Key Laboratory of Medicinal Chemistry and Molecular Diagnosis of Ministry of Education, Key Laboratory of Analytical Science and Technology of Hebei Province, College of Chemistry and Environmental Science, Hebei University, Baoding, China

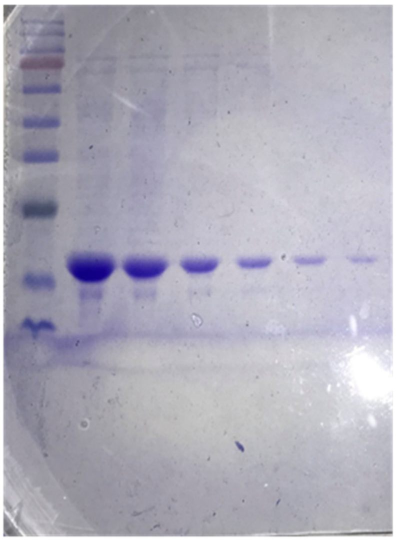

Figure S1. SDS-PAGE result of α-synuclein.

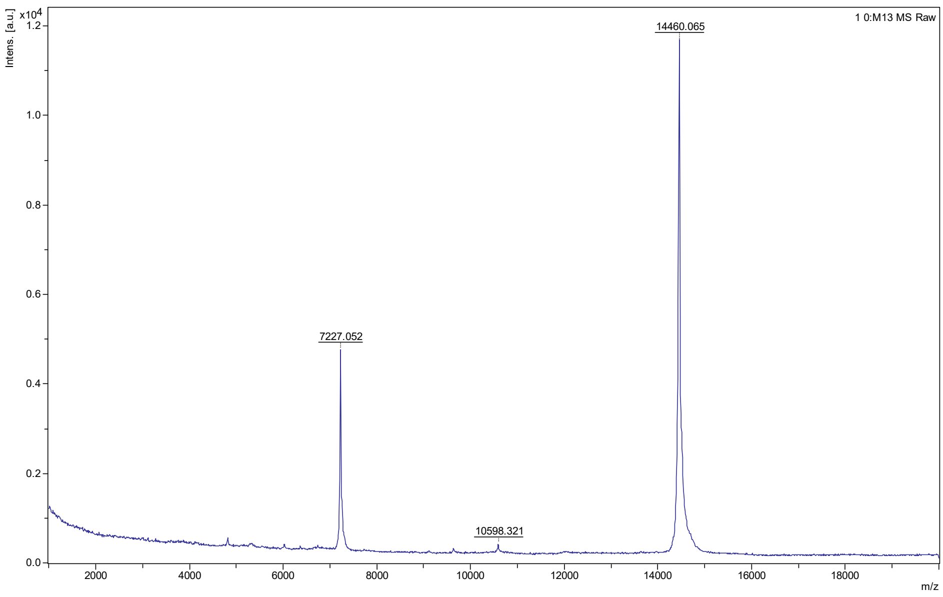

Figure S2. Mass spectrometry (MS) result of α-synuclein. (MALDI-TOF/TOF, Ultraflextreme, Brucker, Germany; α-Cyano-4-hydroxycinnamic acid (CHCA) was chosen as the matrix).

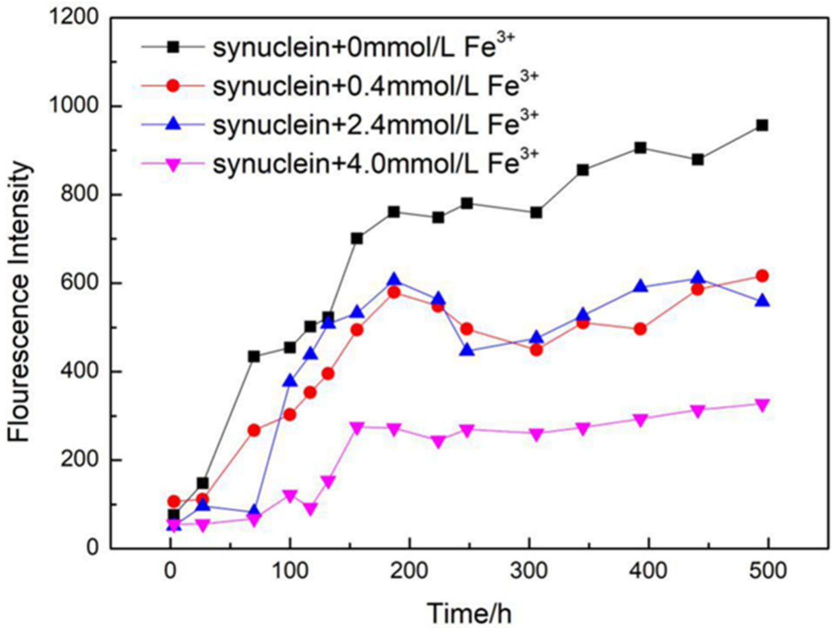

Figure S3. Fibrillation kinetics of α-synuclein under different concentrations of Fe^{3+} by ThT fluorescence assay.

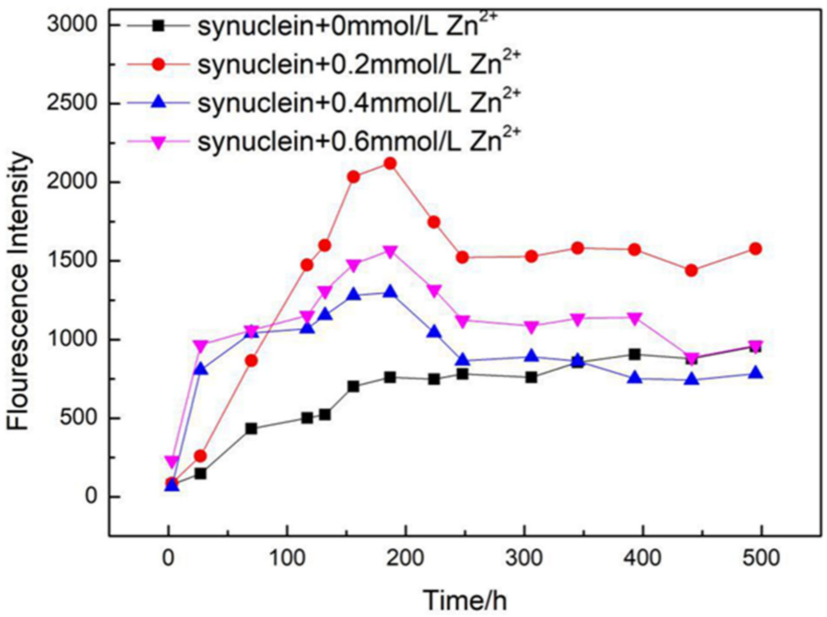

Figure S4. Fibrillation kinetics of α-synuclein under different concentrations of Zn^{2+} by ThT fluorescence assay.

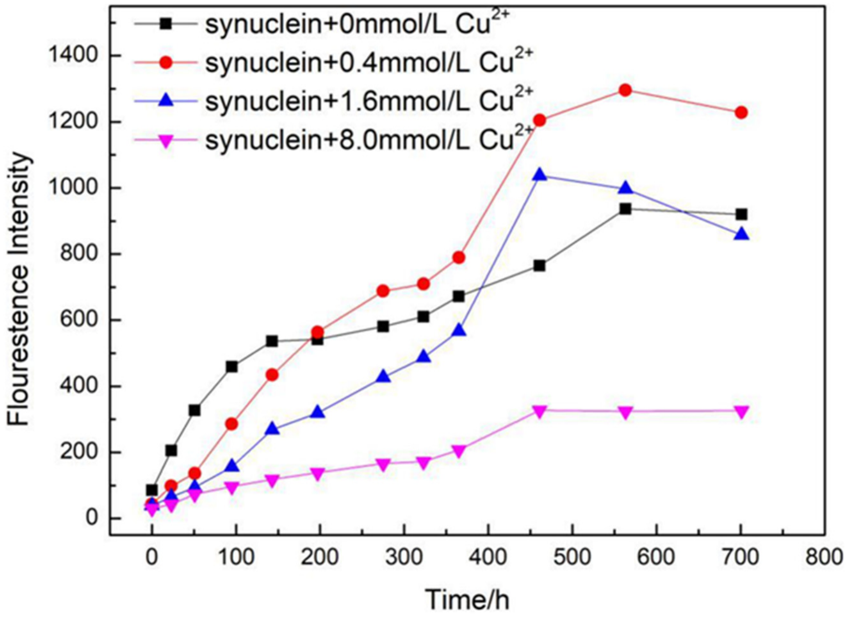

Figure S5. Fibrillation kinetics of α-synuclein under different concentrations of Cu^{2+} by ThT fluorescence assay.

Note: Thioflavin T (ThT) fluorescence assay was used to follow the fibrillation kinetics of α-synuclein. This assay was performed with a Hitachi F-7000 fluorescence spectrophotometer. The parameters were set as follows: excitation wavelength at 450 nm, excitation slit at 5 μm, emission wavelength at 486 nm, emission slit at 10 μm, PMT gain voltage at 700 V. The concentration of ThT solution is 10 μM. The buffer is 20 mM phosphate buffer at pH = 7.4. The assay was performed ex situ. At different time points during α-synuclein fibrillation, 10 μL of incubation solution was taken out of the incubation vial and was added into 1 mL of ThT solution in a 1.0 cm quartz cuvette. The solution in the cuvette was shaken first before each fluorescence measurement.

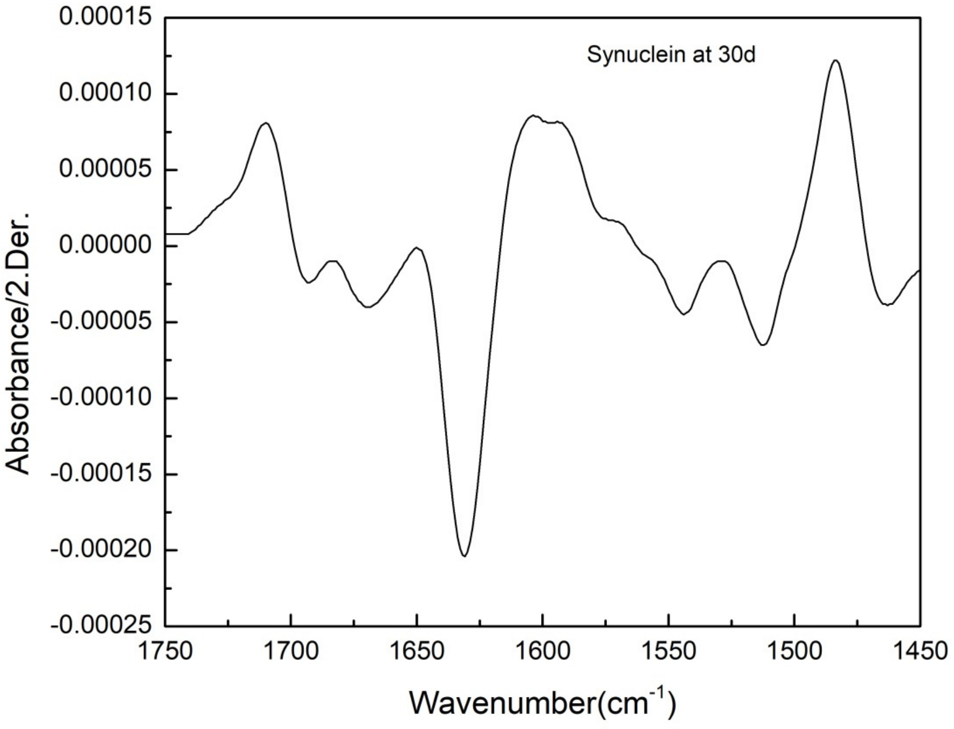

Figure S6. Second derivative spectrum of α-synuclein amyloid fibril in the amide I region.

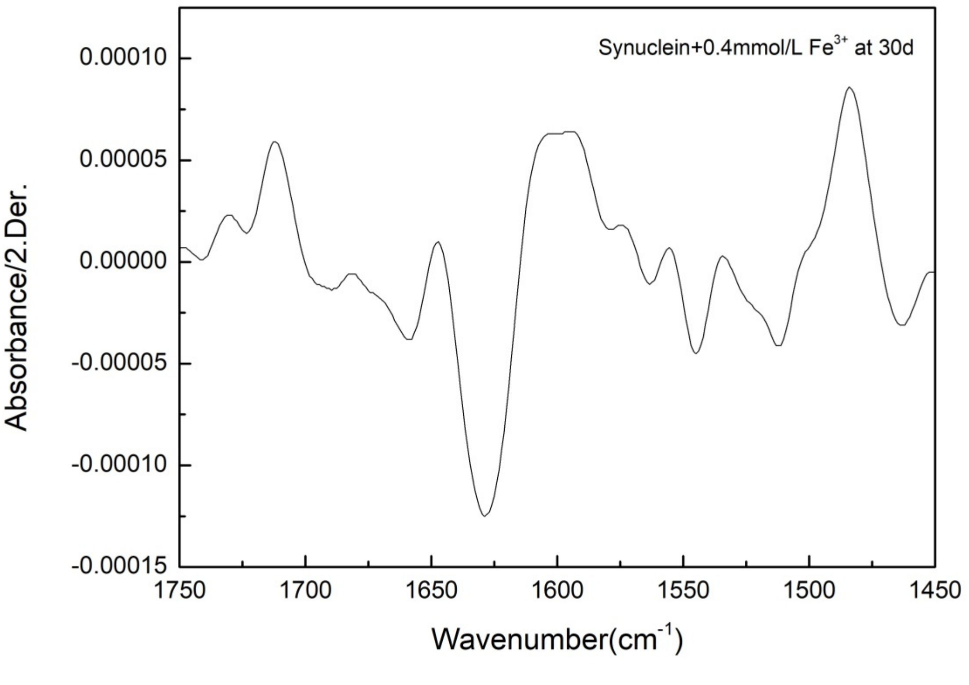

Figure S7. Second derivative spectrum of α-synuclein amyloid fibril under the influence of 0.4 mM Fe^{3+} in the amide I region.

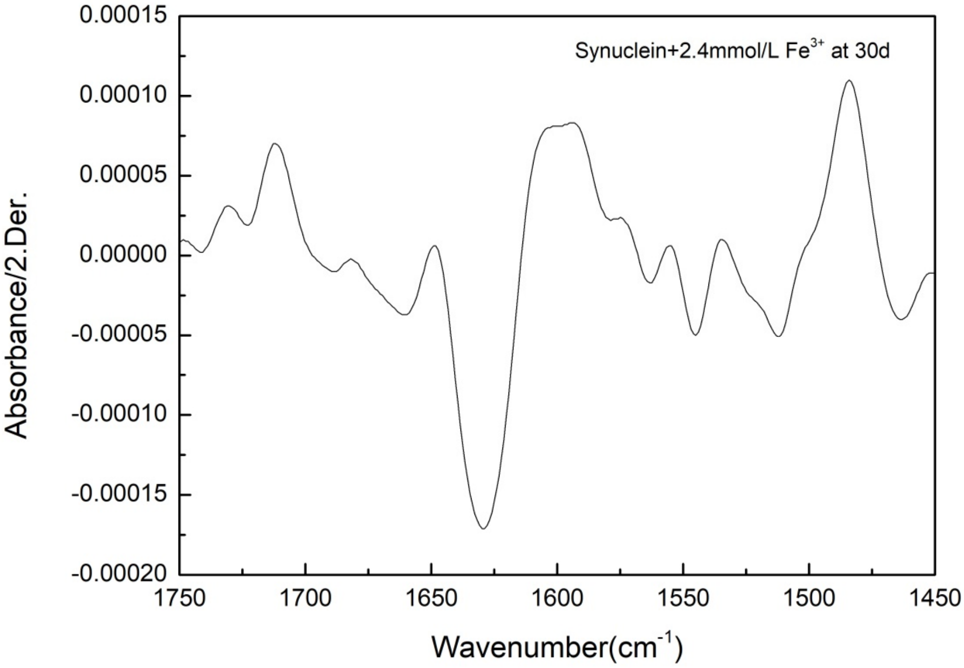

Figure S8. Second derivative spectrum of α-synuclein amyloid fibril under the influence of 2.4 mM Fe^{3+} in the amide I region.

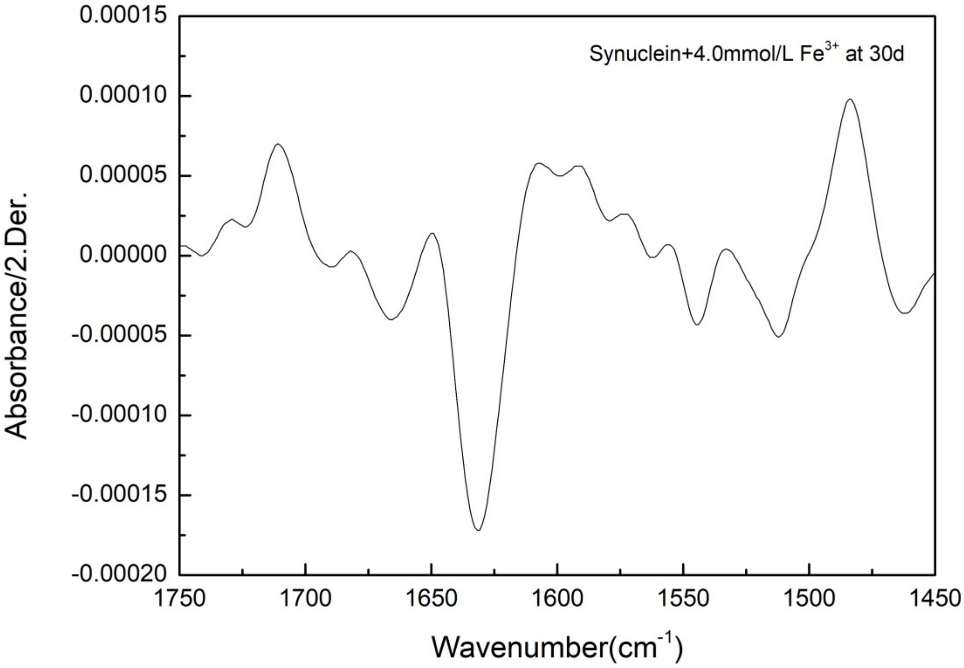

Figure S9. Second derivative spectrum of α-synuclein amyloid fibril under the influence of 4.0 mM Fe^{3+} in the amide I region.

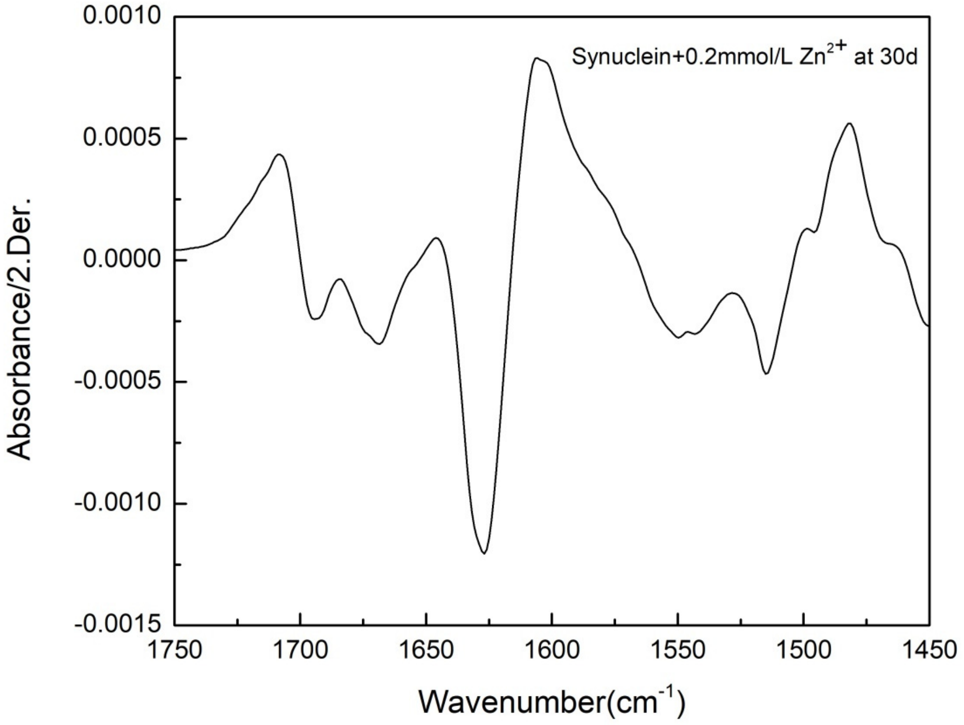

Figure S10. Second derivative spectrum of α-synuclein amyloid fibril under the influence of 0.2 mM Zn^{2+} in the amide I region.

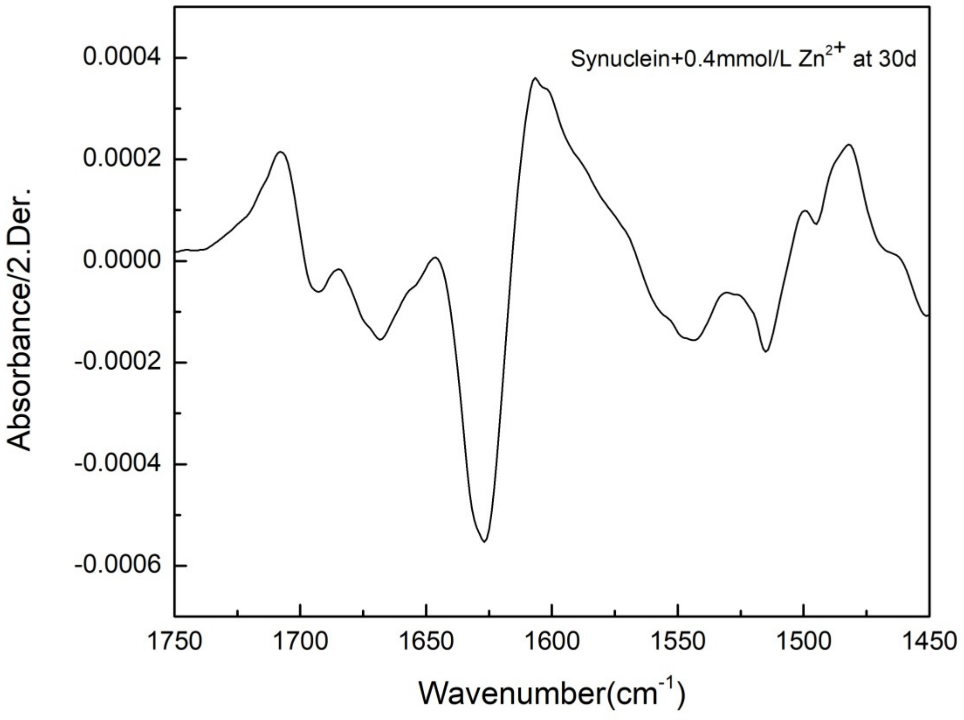

Figure S11. Second derivative spectrum of α-synuclein amyloid fibril under the influence of 0.4 mM Zn^{2+} in the amide I region.

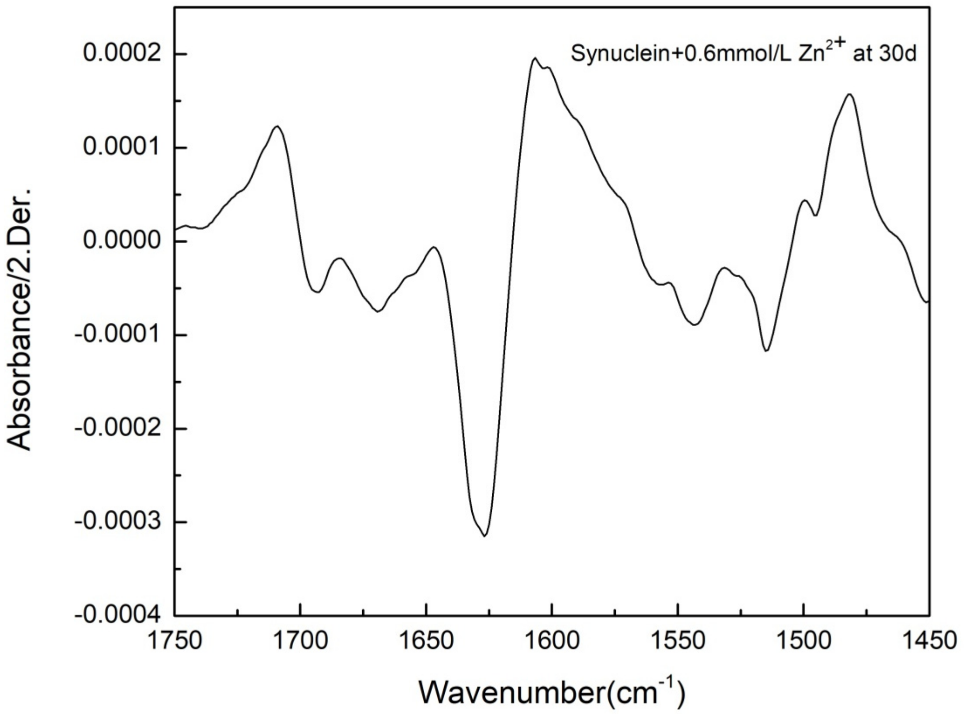

Figure S12. Second derivative spectrum of α-synuclein amyloid fibril under the influence of 0.6 mM Zn^{2+} in the amide I region.

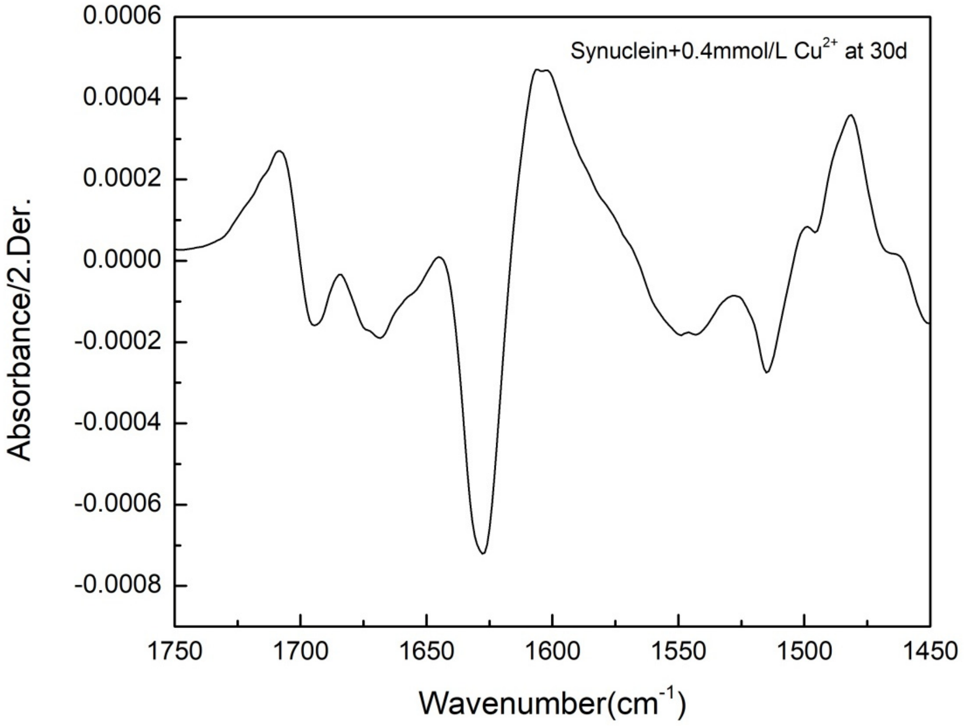

Figure S13. Second derivative spectrum of α-synuclein amyloid fibril under the influence of 0.4 mM Cu^{2+} in the amide I region.

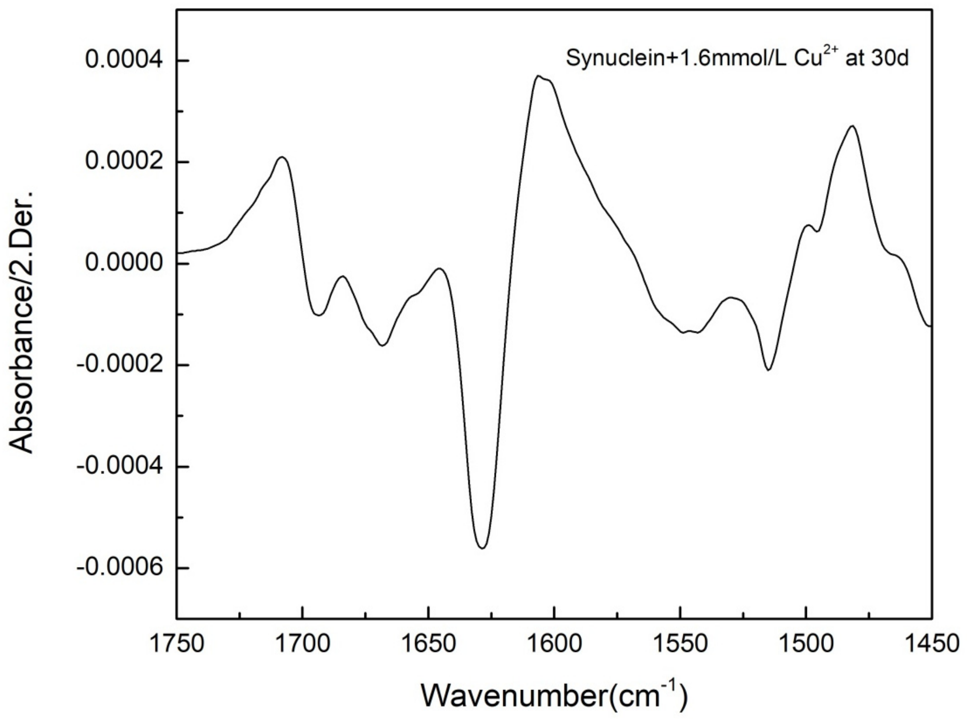

Figure S14. Second derivative spectrum of α-synuclein amyloid fibril under the influence of 1.6 mM Cu^{2+} in the amide I region.

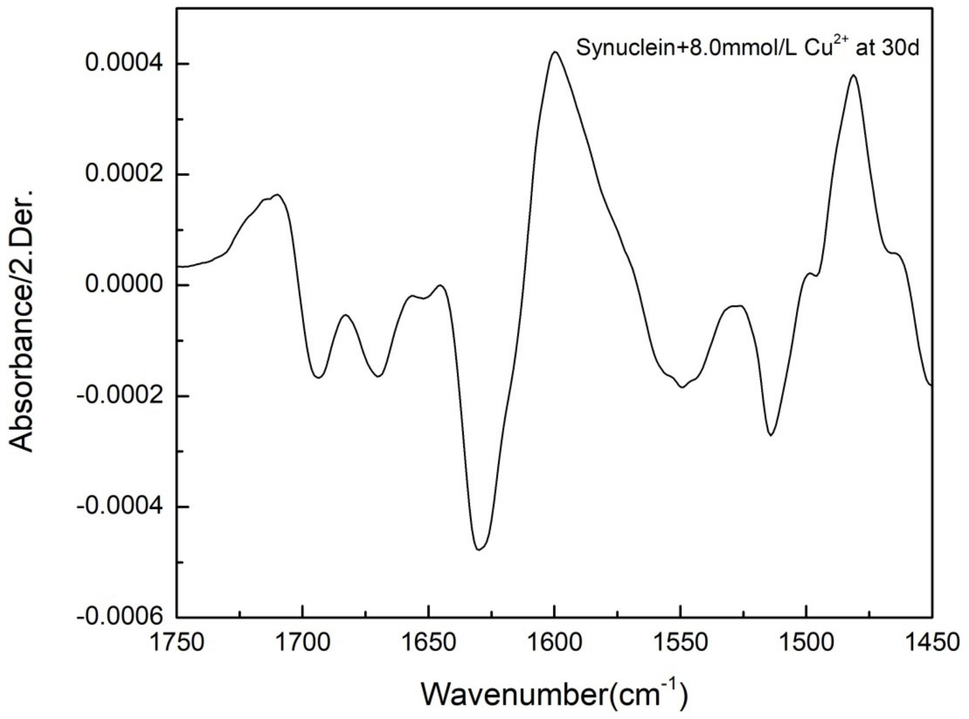

Figure S15. Second derivative spectrum of α-synuclein amyloid fibril under the influence of 8.0 mM Cu^{2+} in the amide I region.

Table. S1. The proportion increase of β-sheet secondary structure in α-synuclein amyloid fibril due to the influences of different concentrations of Fe^{3+}.

Fe^{3+} concentration (mM)	β-sheet (%)	Variation (%)
0	39.5	0
0.4	46.1	16.7
2.4	47.0	19.0
4.0	49.0	24.1

Table. S2. The proportion increase of β-sheet secondary structure in α-synuclein amyloid fibril due to the influences of different concentrations of Zn^{2+}.

Zn^{2+} concentration (mM)	β-sheet (%)	Variation (%)
0	39.5	0
0.2	40.8	3.3
0.4	42.6	7.8
0.6	44.5	12.7

Table. S3. The proportion increase of β-sheet secondary structure in α-synuclein amyloid fibril due to the influences of different concentrations of Cu^{2+}.

Cu^{2+} concentration (mM)	β-sheet (%)	Variation (%)
0	39.5	0
0.4	42.0	6.3
1.6	42.5	7.6
8.0	43.6	10.4

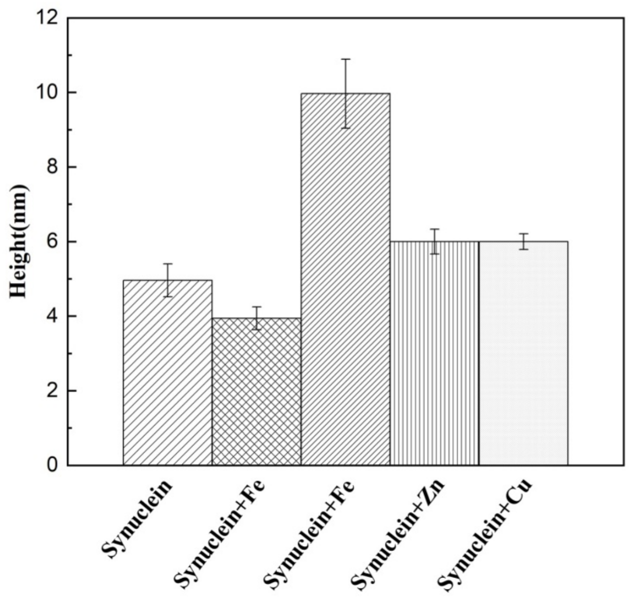

Figure S16. The height analysis of amyloid fibrils of the pristine α-synuclein system, α-synuclein+Fe^{3+} system, α-synuclein+Zn^{2+} system, and α-synuclein+Cu^{2+} system. Note: α-synuclein+Fe^{3+} system has two fibril heights. Each height value and standard deviation was obtained by measuring the fibril height randomly at fifty different locations on the sampled amyloid fibrils.
